# Heterogeneity in the uptake, attendance, and outcomes in a clinical trial of a total diet replacement weight loss programme

**DOI:** 10.1186/s12916-020-01547-4

**Published:** 2020-04-16

**Authors:** Nerys M. Astbury, Kate Tudor, Paul Aveyard, Susan A. Jebb

**Affiliations:** 1 Nuffield Department of Primary Care Health Sciences, Radcliffe Observatory Quarter, University of Oxford, Oxford, OX26GG UK; 2 NIHR Oxford Biomedical Research Centre, Oxford University Hospitals NHS Foundation Trust, Oxford, UK

**Keywords:** Heterogeneity, Weight loss, Total diet replacement, Inequality

## Abstract

**Background:**

Trials have shown total diet replacement (TDR) programmes are safe and effective for weight loss in primary care. However, it is not clear whether participant characteristics affect uptake, attendance, or effectiveness of the programme.

**Methods:**

We used data from 272 trial participants who were invited to participate in a clinical weight loss trial via a letter from their GP. We used a Cochran-Mantel-Haenszel analysis to assess whether accepting an invitation to participate in the trial differed by gender, age, BMI, social deprivation, and the presence of a diagnosis of type 2 diabetes or hypertension. We used mixed generalised linear modelling to examine whether participants’ age, gender, or social deprivation based on area of residence were associated with weight change at 12 months.

**Results:**

Men were less likely to enrol than women (RR 0.59 [95% CI 0.47, 0.74]), and people from the middle and highest BMI tertile were more likely to enrol than those from the lowest tertile (RR 2.88 [95% CI 1.97, 4.22] and RR 4.38 [95% CI 3.05, 6.07], respectively). Patients from practices located in most deprived and intermediate deprived tertiles were more likely to enrol compared with those in the least deprived tertile (RR 1.84 [95% CI 1.81, 2.59] and RR 1.68 [95% CI 1.18, 2.85], respectively). There was no evidence that age or a pre-existing diagnosis of type 2 diabetes (RR 1.10 [95% CI 0.81, 1.50]) or hypertension (RR 0.81 [95% CI 0.62, 1.04]) affected enrolment. In the TDR group, 13% of participants were low engagers, 8% engaged with the weight loss phase only, and 79% engaged in both weight loss and weight maintenance phases of the programme. Those who engaged in the entire programme lost most weight. Subgroup analyses suggested that older participants and those with a higher baseline BMI lost more weight at 1 year than their comparators.

**Conclusion:**

Despite some heterogeneity in the uptake and outcomes of the programme, if the results of this trial are replicated in routine practice, there is no evidence that TDR weight loss programmes would increase inequity.

**Trial registration:**

The DROPLET trial was prospectively registered on ISRCTN registry (ISRCTN75092026).

## Background

Low-energy total diet replacement (TDR) programmes to treat people with obesity involve replacing usual foods with formula products for up to 12 weeks in combination with behavioural support. Evidence from two recent randomised controlled trials shows that offering a TDR programme in primary care is safe and leads to substantial weight loss [1, 2]. However, it is not clear whether participants in such trials are representative of the general practice population of people with obesity. This information is important to indicate the potential of this approach to produce equitable outcomes in routine care.

The current mainstay of weight management support in the UK is referral to a behavioural weight loss programme, typically involving group sessions delivered in the community over 12 weeks [3]. Previous evaluations of these programmes in clinical trials run in routine care settings have reported that women and people who are older and from less deprived areas are more likely to be enrolled [4, 5]. These same trials show no evidence of differences in weight loss in relation to participants’ gender, age, or deprivation, suggesting that participation in these programmes per se does not increase inequalities [6, 7].

Interestingly, the population enrolled in weight loss trials are usually more representative of the general population with obesity than the typical referrals from routine primary care who tend to be middle-aged, older, and nearly all women [8, 9]. The perception that these community weight loss groups cater mainly for middle-aged women may deter practitioners from referring people who do not fit the stereotype, or prevent those who are referred from actually attending.

The TDR programmes tested in recent trials provided one-to-one behavioural support and a diet that involves replacing all meals with specially formulated products. These characteristics may appeal to a different subset of the population than group-based programmes, and it is plausible that not all potential participants will find this approach to weight loss fits with their life equally which could lead to differential outcomes. In this analysis, we explore whether uptake, engagement, and outcomes of a TDR programme offered as part of a clinical trial differed in relation to participant characteristics or the presence of weight-related disease.

## Methods

### Design and setting

The Doctor Referral of Overweight People to Low-Energy total diet replacement Treatment (DROPLET) study was a randomised controlled, two-arm trial to determine the clinical and cost effectiveness of a primary care referral to commercial low-energy TDR programme delivered in the community, compared with usual weight management interventions offered by a practice nurse. Participants in the trial were recruited from ten primary care practices in Oxfordshire, UK. We have published full details of the trial protocol and main results elsewhere [2, 10, 11].

### Recruitment

Each practice searched their electronic records for suitable patients. GPs sent an invitation letter offering the opportunity to take part in a trial comparing two weight management programmes. The letter briefly described the nature of the two weight loss treatments, and that both treatments were offered free of charge as part of the trial, but no details were provided on the location or timing of appointments for either treatment. Interested recipients contacted the research team, and those who met the eligibility criteria were scheduled for an appointment with a nurse at their primary care practice for a formal eligibility check and, if eligible, offered enrolment in the trial. Participants were then randomised in a 1:1 ratio (stratified by general practice and baseline BMI ≤ 35 kg/m^2^ or > 35 kg/m^2^) to either a TDR programme or usual care.

### Treatments

#### TDR intervention

Participants randomised to the intervention group were asked to contact a local counsellor, to arrange regular appointments over 24 weeks. The counsellor provided all TDR products and was trained to provide support for people following a TDR. The appointments took place either at the counsellors’ or at the participants’ home at a mutually convenient time, including weekends and times outside traditional working hours. The sessions comprised motivational support, reassurance, and problem-solving to support initial adherence to the programme and subsequent return to a food-based diet, followed by support for weight loss maintenance. For the first 8 weeks (TDR phase), participants were advised to replace *all* their usual foods and drinks with four of the formula products daily, 750 mL of skimmed milk, 2.25 L of water, or other non-calorific drinks and a fibre supplement providing 810 kcal/day (3389 kJ/day). After 8 weeks, there was a 4-week stepwise reduction in the use of formula products and re-introduction of food-based meals (food re-introduction phase). During the weight maintenance phase (weeks 13–24), participants were advised to consume one formula product a day, with the remainder of diet provided by regular food, and encouraged to attend monthly appointments with the counsellor at 16, 20, and 24 weeks. This weight maintenance phase included a ‘rescue package’, which allowed participants return to the total diet replacement stage for periods of up to 4 weeks if they regained 1 kg or more than their weight at 12 weeks. All consultations and formula products were provided free of charge for the first 24 weeks after which participants were free to choose whether or not to continue with the programme, but at their own cost.

#### Usual care

Participants randomised to the control intervention were offered a behavioural weight management programme provided by a member of the practice team who had been trained to offer a weight loss programme (typically the practice nurse). As part of this programme, participants were provided with a copy of the booklet ‘So you want to lose weight … for good’ [12] and were offered a series of appointments over 12 weeks, at a frequency typically used in the practice (e.g. weekly or bi-weekly).

### Outcome measures

Primary care practices provided the research team with anonymous summary data on gender, age, BMI, and diagnoses of type 2 diabetes and hypertension for all patients who were invited to take part in the trial. The postcode of each practice was used to represent patients’ social deprivation based on the Index of Multiple Deprivation (IMD) [13] for the uptake analysis, and IMD of participants’ home postcodes was used for enrolled participants. The IMD ranks geographical areas of about 500 households in the UK on seven indices: income, employment, health deprivation and disability, education, crime, barriers to housing and services, and living environment. These ranks are grouped into deciles which were used for analysis with lowest decile [1] representing the most deprived areas and highest decile [10] representing the least deprived areas. We examined the presence of diabetes and hypertension alongside demographic characteristics because these are weight-related conditions that often improve on weight loss and this may increase motivation to participate and complete treatment.

During the trial, each of TDR counsellors recorded the number and dates of attendances using a standard record card for each of the participants randomised to the TDR programme. At the end of the trial, counsellors provided a copy of the record card to the research team who determined the number of attendances and allocated engagement in the programme according to the following mutually exclusive patterns of engagement:

- Engaged in entire programme (weight loss and weight maintenance phases) (defined as attending at least 6 out of 8 of the TDR sessions (weeks 1–8) and at least 2 out of 4 of the food re-introduction sessions (weeks 9–12) and at least 2 out of 3 of the weight maintenance sessions (weeks 13–24)).
- Engaged in weight loss phase only (defined as either engaging in the TDR phase only, or engaging in both the TDR and food re-introduction phases but did not engage in the weight maintenance phase)
- Low engagers (did not meet criteria for engaging in any phase)

**Fig. 1:**
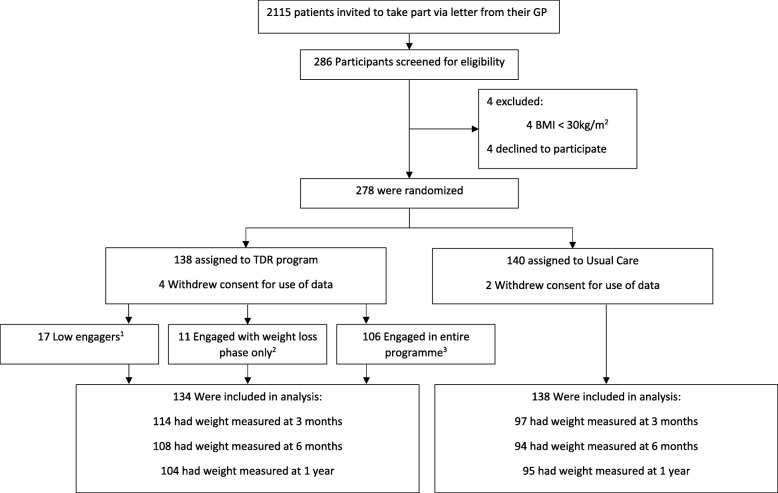
Flow of participants in the DROPLET trial. ^1^Attended < 6 sessions out of the possible 8 sessions during weeks 1–8, < 2 out of the possible 4 sessions offered during weeks 9–12, and < 2 out of the possible 3 sessions offered during weeks 13–26. ^2^Either attended ≥ 6 of the TDR sessions out of the possible 8 during weeks 1–8 only, or attended ≥ 6 of the TDR sessions and ≥ 2 of the food re-introduction sessions out of the possible 4 sessions offered during weeks 9–12. ^3^Engaged in entire programme defined as engaging in weight loss phase (as defined above) and engaging with weight maintenance phase defined as attending ≥ 2 of the possible 3 weight maintenance sessions offered during weeks 13–26

Nine participants did not fall into one of the above groups. These participants were placed into the group reflecting the last phase with which they engaged, irrespective of whether they met our criteria for engagement in prior phases. Participants’ flow through the trial is displayed in Fig. 1.

### Analysis

We split demographic variables and BMI for the invited population into tertiles, and these group cut-points were used in all further analyses. We used the Cochran-Mantel-Haenszel method to calculate adjusted risk ratios (RRs) and 95% confidence intervals (CIs) for demographic characteristics of the enrolled (*n* 272) participants compared with those invited (*n* 2115) but not enrolled (*n* 1843), stratifying by practice. Multivariable logistic regression was used to explore the association between participant characteristics and likelihood of being included in one of the mutually exclusive engagement groups. We used generalised linear mixed effect models to examine whether weight loss at 1 year differed between demographic groups for participants in the TDR group only. Visit and characteristic of interest and the interaction between these variables were used as fixed factors, with other characteristics as additional factors and participant, and practice as random effects with a further covariate for baseline weight.

We compared the difference in weight change from baseline for each level of engagement in the intervention arm with the control group using generalised linear mixed model with visit and engagement group and the interaction between these variables as fixed factors, practice and participant ID as random factors adjusting for age, gender, baseline BMI, IMD decile, diabetes, and hypertension to control confounding. All analyses were exploratory, but we present *p* values to aid interpretation.

## Results

### Characteristics of invited patients and enrolled participants

Across ten practices, 2115 patients were invited to take part in the trial, 286 were screened, and 278 were eligible and enrolled, a recruitment rate of 13%. Of those who were scheduled for a screening visit to obtain consent and assess eligibility, four volunteers declined to participate (thus were not assessed for eligibility) and four were ineligible (BMI < 30 kg/m^2^). Of those 278 enrolled, six people withdraw consent for use of their data after randomisation (four intervention and two control); thus, data from 272 participants (134 intervention and 138 control) were included in this analysis. There was evidence that patients with a BMI > 31.8 ≤ 35.5 kg/m^2^ (RR 2.88 [95% CI 1.97, 4.22]) and those with BMI > 35.5 kg/m^2^ (RR 4.38 [95% CI 3.05, 6.07]) were more likely to enrol than those with BMI ≤ 31.8 kg/m^2^. Men were less likely to enrol compared with women (RR 0.59 [95% CI 0.47, 0.74]). Patients from practices located in the most deprived areas (IMD decile ≤ 6) were more likely to enrol than patients from practices located in the least deprived areas (IMD decile > 8) (RR 1.84 [95% CI 1.81, 2.59]). Patients from practices located in the middle IMD group (IMD decile > 6 ≤ 8) were also more likely to enrol compared with patients from the least deprived areas (RR 1.68 [95% CI 1.18, 2.85]).There was no evidence that age or the presence of a diagnosis of diabetes (RR 1.10 [95% CI 0.81, 1.50]) or hypertension (RR 0.81 [95% CI 0.62, 1.04]) was associated with enrolment (Table 1).

**Table 1:** Characteristics of invited and enrolled populations for all practices

	Invited^1^, *n*	Enrolled, *n* (% of invited)	RR (95% CI)
Gender			
Men	1087	107 (10)	0.59 (0.47, 0.74)*
Women	1022	165 (16)	
Age			
≤ 44 years	732	102 (14)	
> 44 ≤ 56 years	738	103 (14)	1.07 (0.83, 1.34)
> 56 years	643	67 (10)	0.82 (0.62, 1.09)
BMI			
≤ 31.8	703	33 (5)	
> 31.8 ≤ 35.5	697	96 (14)	2.88 (1.97, 4.22)*
> 35.5	689	143 (21)	4.38 (3.05, 6.07)*
IMD Decile			
≤ 6	800	120 (15)	1.84 (1.81, 2.59)*
> 6 ≤ 8	824	112 (14)	1.68 (1.18, 2.85)*
> 8	491	40 (8)	
Type 2 diabetes diagnosis			
Yes	284	41 (14)	1.10 (0.81, 1.50)
No	1831	231 (13)	
Hypertension diagnosis			
Yes	545	63 (12)	0.81 (0.62, 1.04)
No	1570	209 (13)	

*IMD* Index of Multiple Deprivation. IMD is calculated using practice postcode, and lower IMD indicates a more deprived location. *RR* risk ratio

*Statistically significant difference (*p* < 0.01 for χ^2^ test)

^1^Anonymised data on all invited patients were provided by the GP practices for the 2115 invited patients. There was missing data on gender for 6 patients, on age for 2 patients, and on BMI for 26 patients

### Association between participant characteristics and engagement with the programme

Baseline characteristics for the 134 participants randomised to the TDR group and for whom data were available for analysis are presented in Table 2. Seventeen (13%) participants did not engage with the programme (attended < 6 sessions out of 8 during the TDR phase (weeks 1–8), and < 2 out of 4 session during the food re-introduction phase (weeks 9–12) and < 2 out of 3 sessions offered during the weight maintenance phase (weeks 13–24)). Eleven participants (8%) engaged only in the weight loss phase (attended ≥ 6 sessions out of 8 during the TDR phase (weeks 1–8) or ≥ 6 sessions during the TDR phase and ≥ 2 of 4 sessions offered during the food re-introduction phase (weeks 9–12)), and 106 participants (79%) engaged in the weight maintenance phase (attended ≥ 2 out of three sessions offered during weight maintenance phase (week 13–26)) in addition to the weight loss phase as described above. There was no evidence that participant characteristics were associated with engagement (Table 3).

**Table 2:** Baseline characteristics of recruited participants who were randomised to the TDR intervention

	**N**	**Mean**	**SD**
Age (years)	134	48.2	11.5
Weight (kg)	134	107.9	18.9
Height (cm)	134	169.2	9.5
BMI	134	37.6	5.7
IMD decile	134	7.7	2.0
		**N (%)**	
Gender			
Men		53 (39.6)	
Women		81 (60.5)	
Ethnicity			
White British		121 (90.3)	
Other		13 (9.7)	
Type 2 diabetes diagnosis		21 (15.7)	
Hypertension diagnosis		33 (24.6)	

*BMI* body mass index

**Table 3:** Engagement of the participants enrolled into the DROPLET trial and randomised to the TDR intervention

	Low engagers^1^				Engaged in weight loss phase only^2^				Engaged in weight loss and weight maintenance phases^3^			
	*N* (%)	OR	95% CI	*p*	*N* (%)	OR	95% CI	*p*	*N* (%)	OR	95% CI	*p*
Total	17 (13)				11 (8)				106 (79)			
Gender												
Men	6 (35)	0.89	0.29, 2.66	0.823	3 (27)	0.67	0.14, 3.16	0.618	44 (42)	1.33	0.68, 1.89	0.56
Women	11 (65)				8 (72)				62 (58)			
Age												
≤ 44 years	7 (41)				7 (64)				34 (32)			
> 44 ≤ 56 years	6 (35)	1.27	0.36, 4.52	0.728	3 (27)	0.68	0.09, 2.69	0.405	41 (39)	1.19	0.64, 2.19	0.38
> 56 years	4 (24)	1.11	0.36, 5.84	0.881	1 (9)	0.22	0.02, 2.30	0.208	31 (29)	1.60	0.78, 3.17	0.46
IMD decile												
≤ 6	6 (35)	2.00	0.51, 7.89	0.322	2 (18)	0.72	0.09, 5.80	0.760	30 (28)	0.62	0.18, 2.14	0.45
> 6 ≤ 8	5 (29)	1.17	0.23, 2.82	0.814	6 (55)	2.75	0.56, 13.57	0.213	33 (31)	0.563	0.18, 1.58	0.26
> 8	6 (35)				3 (27)				43 (41)			
Baseline BMI												
≤ 31.8 kg/m^2^	2 (12)				0				15 (14)			
> 31.8 ≤ 35.5 kg/m^2^	3 (18)	0.51	0.07, 3.49	0.494	0	–	–	–	37 (35)	2.11	0.31, 14.44	0.45
> 35.5 kg/m^2^	12 (71)	1.51	0.29, 7.81	0.626	11 (100)	–	–	–	54 (51)	0.31	0.06, 1.56	0.60
Type 2 diabetes												
Yes	16 (94)	0.22	0.04, 2.44	0.178	1 (9)	0.53	0.05, 5.30	0.760	19 (18)	3.69	0.70, 19.39	0.12
No	1 (6)				10 (91)				87 (82)			
Hypertension												
Yes	14 (82)	0.83	0.17, 2.72	0.806	1 (9)	0.39	0.04, 3.95	0.426	28 (26)	1.57	0.43, 5.75	0.49
No	3 (18)				10 (91)				78 (74)			

^1^Attended < 6 sessions out of the possible 8 sessions during weeks 1–8, < 2 out of the possible 4 sessions offered during weeks 9–12, and < 2 out of the possible 3 sessions offered during weeks 13–26

^2^Attended ≥ 6 sessions out of the possible 8 sessions during weeks 1–8 only or attended ≥ 6 sessions out of the possible 8 sessions during weeks 1–8 and ≥ 2 out of the possible 4 sessions offered during weeks 9–12

^3^Attended 2 out of the possible 3 sessions offered during weeks 13–26

### Associations between participant characteristics and weight loss

**Fig. 2:**
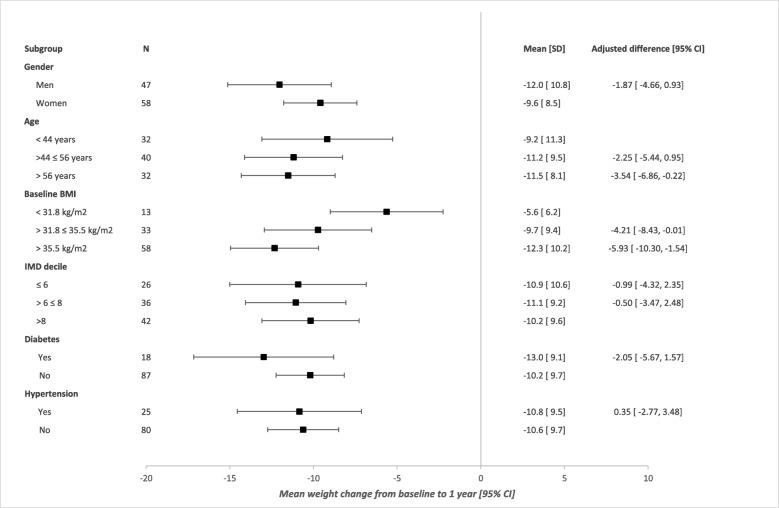
Subgroup differences in weight change from baseline at 1 year. Cut points for tertile splits determined from the invited population. IMD, Index of Multiple Deprivation decile calculated using practice postcode; lower IMD indicates a more deprived location

There was no evidence of any difference between men and women for weight loss at 1 year (mean difference − 1.87 kg [95% CI − 4.66, 0.93], *p* = 0.191). There was evidence that the oldest participants (aged > 56 years) lost more weight compared with the youngest participants (aged ≤ 44 years) (mean difference − 3.54 [95% CI − 6.86, 0.22], *p* = 0.037) and that participants with baseline BMI (318–35.5) and those with highest baseline BMI (> 35.5 kg/m^2^) lost more weight compared with the group of lowest BMI (≤ 31.8 kg/m^2^) (mean differences − 4.21 [95% CI − 8.43, − 0.01], *p* = 0.05, and − 5.93 kg [95% CI − 10.3, − 1.54], *p* = 0.008, respectively). However, there was no evidence that after adjusting for all other factors, weight loss at 1 year differed by area of residence or between those with pre-existing diagnosis of type 2 diabetes or hypertension and their peers (Fig. 2).

### Association between pattern of engagement and weight change

**Fig. 3:**
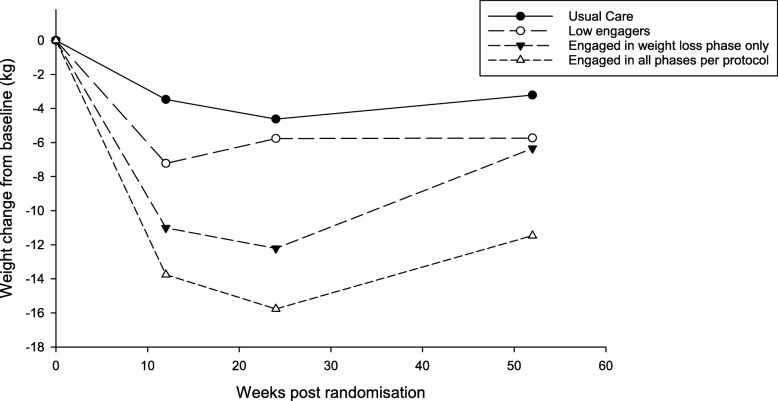
Weight trajectories according to pattern of engagement. Usual care: offered usual care weight management. Low engagers: attended < 6 sessions out of the possible 8 sessions during weeks 1–8, < 2 out of the possible 4 sessions offered during weeks 9–12, and < 2 out of the possible 3 sessions offered during weeks 13–26. Engaged in weight loss phase only: either attended ≥ 6 of the TDR sessions out of the possible 8 during weeks 1–8 only, or attended ≥ 6 of the TDR sessions and ≥ 2 of the food re-introduction sessions out of the possible 4 sessions offered during weeks 9–12. Engaged in all phases as per protocol: engaged in entire programme defined as engaging in weight loss phase (as defined above) and engaging with weight maintenance phase defined as attending ≥ 2 of the possible 3 weight maintenance sessions offered during weeks 13–26

The adjusted weight losses for the control group and the TDR group split by each group of engagement as well as any differences between groups are presented in Table 4 and Fig. 3**.** Participants who engaged in all phases of the programme lost more weight than the control group at 3 and 6 months and 1 year. Weight loss in the participants who engaged with both the TDR and food re-introduction phases, but not weight maintenance phase, was significantly greater than the control group at 3 and 6 months, but there was no evidence of any difference from the control group at 1 year. There was no difference in weight change between people randomised to the intervention group who did not engage in the TDR phase and the control group.

**Table 4:** Weight change across different groups of engagement with the TDR treatment

Attendance group	*N*	Adjusted mean weight change from baseline (kg) (95% confidence intervals)			Within group, *p* value compared with baseline			Between group, *p* value compared with control		
		3 months	6 months	1 year	3 months	6 months	1 year	3 months	6 months	1 year
Control (usual care)	138	− 3.47 (− 4.53, − 2.41)	− 4.62 (− 5.69, − 3.54)	− 3.21 (− 4.27, – 2.14)	< 0.001	< 0.001	< 0.001	–	–	–
Low engagers^1^	17	− 7.22 (− 11.9, − 2.56)	− 5.76 (− 9.43, − 2.10)	− 5.73 (− 9.20, − 2.26)	0.005	0.006	0.004	0.177	0.727	0.271
Engaged with weight loss phase only^2^	11	− 11.01 (− 14.24, − 7.78)	− 12.22 (− 15.74, − 8.70)	− 6.34 (− 10.05, − 2.63)	< 0.001	< 0.001	< 0.001	< 0.001	< 0.001	0.266
Engaged in entire programme (per protocol)^3^	106	− 13.76 (− 14.82, − 12.69)	− 15.77 (− 14.82, − 12.70)	− 11.47 (− 12.58, − 10.37)	< 0.001	< 0.001	< 0.001	< 0.001	< 0.001	< 0.001

^1^Did not engage in TDR phase, food re-introduction phase, or weight maintenance phase (attended < 6 sessions out of the possible 8 sessions during weeks 1–8, < 2 out of the possible 4 sessions offered during weeks 9–12, and < 2 out of the possible 3 sessions offered during weeks 13–26)

^2^Engaged with weight loss phase (either attended ≥ 6 of the TDR sessions out of the possible 8 during weeks 1–8 or attended ≥ 6 of the TDR sessions and ≥ 2 of the food re-introduction sessions out of the possible 4 sessions offered during weeks 9–12)

^3^Engaged in entire programme defined as engaging in weight loss phase (as defined above) and engaging with weight maintenance phase defined as attending ≥ 2 of the possible 3 weight maintenance sessions offered during weeks 13–26

## Discussion

People with a higher BMI, women, and those from areas of greater socioeconomic deprivation were more likely to enrol in a clinical trial investigating the effectiveness of a TDR programme. However, there was little evidence to suggest that age or the presence of a pre-existing diagnosis of either hypertension or diabetes was associated with enrolment. For participants randomised to the TDR programme, there was no evidence that age, gender, BMI, socioeconomic deprivation, or the presence of either pre-existing diabetes or hypertension diagnoses were associated with the likelihood of engaging in the programme. Participants who engaged with all phases of the programme lost the most weight. Older people and those with a higher baseline BMI had greater weight loss at 1 year.

Some factors may have affected the associations we observed. First, in the analysis of uptake, we used the IMD of the practice area as the indicator of a patient’s own level of deprivation, but an individual may be more or less deprived than the average person in the area in which they live. This is likely to introduce random error into the association, which usually underestimates the strength of the true association and therefore is unlikely to explain why people from more deprived areas appear more likely to participate. We cannot exclude the possibility that less deprived people from more deprived areas or more deprived people from less deprived areas were most likely to enrol. Secondly, this was an exploratory analysis; the study was not planned to detect these associations, so it is possible that we failed to detect associations due to a lack of statistical power. Definitions of engagement were not pre-specified, and a small number of patients had an inconsistent attendance pattern that could not be classified into our groupings. Thirdly, the data come from a clinical trial, where people may respond differently to a situation in which they were offered the same programme as part of routine care, perhaps because the treatment is perceived as unproven or because of the need for additional appointments to take part in trial assessments.

Although we had data on ethnicity, the overwhelming majority of participants in the study were White British, meaning that it was not possible to assess whether uptake, completion, or weight loss varied by ethnicity. Furthermore, although the sample was representative of the local population, the area where the study took place is a more affluent and less ethnically diverse population than the UK as a whole [14, 15]. Further evidence on the acceptability of the TDR approach in more deprived areas with a greater proportion of black and minority ethnic participants is needed.

Men were less likely to enrol in this trial of a TDR weight loss programme than women, a phenomenon which has also been observed in trials of community weight loss groups, and in treatments for obesity generally [6, 7]. However, the disparity by gender was less than has been reported in trials of these other programmes [5], perhaps suggesting that men found the TDR programme more acceptable than community weight loss groups, which has also been noted in a recent trial testing a similar weight loss programme [16]. There was no evidence of a difference in weight loss by gender as reported in a trial of a community weight loss group [6]. We found no evidence that age influenced enrolment in this trial, but older people lost more weight. This is in contrast with a trial of primary care referral to community weight loss groups, where older people were more likely to enrol compared with their younger counterparts [5]. Uptake was higher in those with a higher starting BMI, perhaps because those with the most weight to lose are more attracted by an intensive weight loss programme. Arguably, patients with a pre-existing diagnosis of hypertension and/or type 2 diabetes would have the most to gain from losing weight and might therefore have been more motivated to do so [17], but there was no evidence of greater uptake, engagement, or weight loss in this group and the confidence intervals exclude large though not moderate sized associations. It has been suggested that people with type 2 diabetes tend to lose less weight than people who do not have diabetes, but we found no evidence of this [18].

Examining the socioeconomic equity of this kind of intervention is important since motivation, organisation, and capacity, including material resources, are required to enact the behavioural responses necessary to lose weight and concern has been expressed that interventions, such as a TDR programme, which require a high level of agency, may widen socioeconomic inequalities in health [19]. There is some evidence that those from more deprived areas are less likely to enrol in community weight loss groups [20]. However, in this trial of a TDR programme, those from the most deprived areas were *more* likely to accept the invitation to participate in the trial and weight loss outcomes did not differ in relation to deprivation. Accordingly, we find no evidence to suggest that if the present findings are replicated when offered as part of routine care, TDR weight loss programmes would increase inequity. It is possible that some patients will have known that these programmes cost around £700 if paid for privately, and this may have created a bigger incentive to participate for people with more limited financial resources. Alternatively, it may be due to the one-to-one support offered at a time and place to suit the participant, which might make it easier to engage with TDR programmes than in weight loss groups which are run at a fixed time and location.

Weight change in people who had low engagement with the programme was not significantly different from the control group. People who engaged with the weight loss phase only up to the end of food re-introduction at 12 weeks but did not engage with the weight maintenance phase of the programme lost more weight at 3 and 6 months than the control group. However, these participants gained weight more rapidly between 6 months and 1 year than those who continued to engage with the weight loss maintenance phase and their weight was not significantly different from the control group at 1 year. This apparent benefit of continued ‘engagement’ may reflect ongoing contact and support, or some specific component of the later phase of the behavioural programme which helps to develop longer-term weight maintenance skills. But, the large majority of people completed all phases of the study, so the estimates of the effect of short-term ‘weight loss only’ were imprecise and further data is required to fully examine the effect of short courses of TDR for weight loss with no further support for weight maintenance. It is also plausible that the apparent differences between groups may reflect greater commitment of these individuals to their weight loss attempt, rather than the extended support offered by the programme, and it is not possible to draw conclusions about optimal programme duration from this analysis.

## Conclusion

There were modest differences in uptake of a total diet replacement programme and subsequent weight loss in relation to participant characteristics. However, there is no evidence from this trial that if TDR weight loss programmes were offered in routine care, it would increase inequity.

## Data Availability

The datasets used and/or analysed during the current study are available from the corresponding author on reasonable request.

## Abbreviations

BMI: Body mass index
CI: Confidence interval
DROPLET: Doctor Referral of Overweight People to Low-energy total diet replacement Treatment
IMD: Index of Multiple Deprivation
NHS: National Health Service
RR: Risk ratio
TDR: Total diet replacement

## References

[1] Lean M, Brosnahan N, McLoone P, McCombie L, Higgs AB, Ross H (2013). Feasibility and indicative results from a 12-month low-energy liquid diet treatment and maintenance programme for severe obesity. Br J Gen Pract.

[2] Astbury NM, Aveyard P, Nickless A, Hood K, Corfield K, Lowe R (2018). Doctor referral of overweight people to low energy total diet replacement treatment (DROPLET): pragmatic randomised controlled trial. BMJ..

[3] National Institute for Health and Clinical Excellence (NICE). Obesity: identification, assesment and management London; 2014.

[4] Pagoto SL, Schneider KL, Oleski JL, Luciani JM, Bodenlos JS, Whited MC (2012). Male inclusion in randomized controlled trials of lifestyle weight loss interventions. Obesity (Silver Spring).

[5] Ahern AL, Aveyard P, Boyland EJ, Halford JC, Jebb SA, Team W (2016). inequalities in the uptake of weight management interventions in a pragmatic trial: an observational study in primary care. Br J Gen Pract.

[6] Ahern AL, Wheeler GM, Aveyard P, Boyland EJ, Halford JCG, Mander AP (2017). Extended and standard duration weight loss referrals for adults in primary care (WRAP): a pragmatic randomised controlled trial. Lancet.

[7] Jebb SA, Ahern AL, Olson AD, Aston LM, Holzapfel C, Stoll J (2011). Primary care referral to a commercial provider for weight loss treatment versus standard care: a randomised controlled trial. Lancet..

[8] Stubbs RJ, Pallister C, Whybrow S, Avery A, Lavin J (2011). Weight outcomes audit for 34,271 adults referred to a primary care/commercial weight management partnership scheme. Obes Facts.

[9] Ahern AL, Olson AD, Aston LM, Jebb SA (2011). Weight watchers on prescription: an observational study of weight change among adults referred to weight watchers by the NHS. BMC Public Health.

[10] Kent S, Aveyard P, Astbury N, Mihaylova B, Jebb SA (2019). Is doctor referral to a low-energy total diet replacement program cost-effective for the routine treatment of obesity?. Obesity (Silver Spring).

[11] Jebb SA, Astbury NM, Tearne S, Nickless A, Aveyard P (2017). Doctor referral of overweight people to a low-energy treatment (DROPLET) in primary care using total diet replacement products: a protocol for a randomised controlled trial. BMJ Open.

[12] British Heart Foundation (2005). So you want to lose weight... for good.

[13] Department for Communities and Local Government. The English indicies of Deprivation 2015 [Available from: https://www.gov.uk/government/statistics/english-indices-of-deprivation-2015. Accessed 6 Nov 2019.

[14] Department for Communities and Local Government. The English Indices of Deprivation 2015 Statistical Release 2015.

[15] Office for National Statistics. 2011 Census analysis: Ethnicity and religion of the non-UK born population in England and Wales: 2011. 2015.

[16] Taylor R, Leslie WS, Barnes AC, Brosnahan N, Thom G, McCombie L (2018). Clinical and metabolic features of the randomised controlled diabetes remission clinical trial (DiRECT) cohort. Diabetologia..

[17] Whitlock GL, Sherliker S, Clarke P, Emberson R, Halsey J, Qizilbash J, Collins N, Peto R, Prospective Studies Collaboration (2009). Body-mass index and cause-specific mortality in 900 000 adults: collaborative analyses of 57 prospective studies. Lancet..

[18] Anderson JW, Kendall CW, Jenkins DJ (2003). Importance of weight management in type 2 diabetes: review with meta-analysis of clinical studies. J Am Coll Nutr.

[19] White M, Adams J, Heywood P, Babones S (2009). How and why do interventions that increase health overall widen inequalities within populations?. Social Inequalities and Public Health: Policy Press Scholarship Online.

[20] Relton C, Li J, Strong M, Holdsworth M, Cooper R, Green M (2014). Deprivation, clubs and drugs: results of a UK regional population-based cross-sectional study of weight management strategies. BMC Public Health.

